# A Smartphone-Based Low-Cost Inverted Laser Fluorescence Microscope for Disease Diagnosis

**DOI:** 10.3390/bios12110960

**Published:** 2022-11-02

**Authors:** Omar Ormachea, Alex Villazón, Patricia Rodriguez, Mirko Zimic

**Affiliations:** 1 Centro de Investigaciones Opticas y Energías (CIOE), Universidad Privada Boliviana (UPB), Cochabamba, Bolivia; 2 Centro de Investigaciones en Nuevas Tecnologías Informáticas (CINTI), Universidad Privada Boliviana (UPB), Cochabamba, Bolivia; 3 Instituto de Investigaciones Biomédicas (IIBISMED), Facultad de Medicina, Universidad Mayor de San Simón (UMSS), Cochabamba, Bolivia; 4 Laboratorio de Bioinformática, Biología Molecular y Desarrollos Tecnológicos, Facultad de Ciencias y Filosofía, Universidad Peruana Cayetano Heredia (UPCH), Lima, Peru

**Keywords:** low-cost fluorescence microscopy, smartphone microscopy, 3D-printed devices, laser-based microscopy

## Abstract

Fluorescence microscopy is an important tool for disease diagnosis, often requiring costly optical components, such as fluorescence filter cubes and high-power light sources. Due to its high cost, conventional fluorescence microscopy cannot be fully exploited in low-income settings. Smartphone-based fluorescence microscopy becomes an interesting low-cost alternative, but raises challenges in the optical system. We present the development of a low-cost inverted laser fluorescence microscope that uses a smartphone to visualize the fluorescence image of biological samples. Our fluorescence microscope uses a laser-based simplified optical filter system that provides analog optical filtering capabilities of a fluorescence filter cube. Firstly, we validated our inverted optical filtering by visualizing microbeads labeled with three different fluorescent compounds or fluorophores commonly used for disease diagnosis. Secondly, we validated the disease diagnosis capabilities by comparing the results of our device with those of a commercial fluorescence microscope. We successfully detected and visualized Trypanosoma cruzi parasites, responsible for the Chagas infectious disease and the presence of Antineutrophil cytoplasmic antibodies of the ANCA non-communicable autoimmune disease. The samples were labeled with the fluorescein isothiocyanate (FITC) fluorophore, one of the most commonly used fluorophores for disease diagnosis. Our device provides a 400× magnification and is at least one order of magnitude cheaper than conventional commercial fluorescence microscopes.

## 1. Introduction

Fluorescence microscopy and the use of fluorescent molecules (i.e., fluorophores) in biology is a powerful tool in healthcare [1]. Although commercial fluorescent microscopes are widely available, their high costs (mainly due to sophisticated optics for light filtering) strongly limit their use in low-income settings.

Smartphone microscopy [2] has become an interesting alternative, enabling more compact, affordable, and flexible solutions compared to conventional microscopy. However, many microscopic techniques in the area of disease diagnosis require a minimum optical magnification of 400× (for the direct observation of microorganisms through the microscope’s ocular), often not reached by this type of low-cost microscope.

Low-cost fluorescence microscopy [3,4,5,6,7,8,9,10] raises several challenges, mainly in the optical system, which requires special filtering of the light sources (e.g., LEDs or lasers) used to excite the fluorophores, while keeping the filtering system affordable, notably avoiding the use of costly fluorescence microscope cubes from conventional fluorescence microscopy (based on dichroic mirrors and special filters).

Fluorescence microscopy is an effective procedure for the diagnosis of many infectious and non-communicable diseases [1]. It is often the only way to make a diagnosis. Many microorganisms (e.g., parasites, bacteria) can be specifically detected and identified through fluorescence labeling. The use of fluorochrome-labeled antibodies facilitates this identification, increasing detection sensitivity and improving specificity with respect to white light optical microscopy.

For disease diagnosis, low-cost fluorescence microscopy can be seen as an interesting alternative for low-income countries, where several endemic diseases (e.g., Chagas and tuberculosis) affect millions of people, and are also spreading in non-endemic areas, such as Europe, USA, Canada, and Japan, mainly through migration [11,12]. In addition, several non-communicable diseases, e.g., autoantibodies and autoimmune diseases [1], are not detected in timely fashion due to the lack of fluorescence microscopes in low-income settings.

In general, disease diagnosis requires the detection and identification of microorganisms (e.g., parasites, bacteria, or cells) through fluorescence labeling. In [9] a low-cost fluorescence microscope is used to detect Mycobaterium tuberculosis, using auramine fluorophore [13], which reduces the required 1000× magnification for conventional non-fluorescence techniques to only 400×. Unfortunately, because of the complexity of the optical filtering system, other low-cost fluorescence microscopes [3,4,5,6,7,8,10] do not reach the required magnification for the diagnosis of 400×: they are either a proof-of-concept (used to identify fluorescent particles) or are limited to the visualization of cells, but cannot be used for diagnosis.

In this article, we present a smartphone-based low-cost inverted fluorescence microscope that has a simplified optical filter system, can reach at least a 400× magnification, and can be used for disease diagnosis (infectious and non-communicable diseases). Our hypothesis is that it is possible to replace the costly optical fluorescence filter cubes with a simplified filter based on a low-cost laser and a cheap barrier filter. Our microscope uses an inverted optical scheme allowing for a direct excitation signal through the sample instead of the common epi-illumination. This avoids the need for a dichroic mirror and an excitation filter due to the narrow bandwidth of the laser excitation signal.

Our proposed device is compatible with all diagnostic kits that use fluorescein isothiocyanate (FITC) as labeling dye [14,15]. FITC is one of the more commonly used fluorophores for diagnosis due to its high absorption, excellent fluorescence quantum yield, affordable cost, and good water solubility [16].

Instead of using the standard 488 nm wavelength for the excitation signal, used by the conventional fluorescence microscope (which is closer to the maximum excitation absorption peak of the FITC fluorophore at 495 nm), our inverted microscope uses a low-cost 405 nm laser source, resulting in a much lower absorption. Our hypothesis is that, since we use a focalized high-intensity laser source, this will compensate for the low-level absorption of FITC, thus obtaining fluorescence images similar to those of a conventional fluorescence microscope.

The validation of our proposed device was performed in two ways:

- We validated the proposed low-cost laser-based simplified filtering system by using microbeads labeled with three fluorophores commonly used for disease diagnosis (i.e., FITC, PE, and PE-Cy5).
- We validated the diagnosis capability of our device by using biological samples of two diseases (one infectious and one non-communicative) and comparing the obtained results with those of a conventional fluorescence microscope. The samples were labeled with an FITC fluorophore.

For the diagnosis of an infectious disease, we chose Chagas, an endemic disease that affects millions of people, mainly in Latin America, is caused by the protozoan parasite Trypanosoma cruzi (T. cruzi), and is transmitted in the endemic areas during a bite of a hematophagous insect belonging to the Triatominiae family. In non-endemic areas, the transmission occurs mainly by blood transfusion, organ transplant from infected people, or vertical transmission from mother to child [11]. The diagnosis of Chagas by immunofluorescence requires the visualization of fixed forms of T. cruzi apimastigotes, indirectly labeled with a fluorophore (e.g., FITC or Alexa Fluor 488). This visualization of the fluorescent T. cruzi parasite requires a magnification of at least 400×.

For the diagnosis of a non-communicable disease, we chose the ANCA test. Anti-neutrophil cytoplasm antibodies (ANCA) are autoantibodies that mainly target antigens present in cytoplasmic granules of neutrophils, an autoimmune disease affecting small blood vessels in the body [17]. The diagnosis of this disease was conducted by observing granules on fixed neutrophils after indirect marking with the FITC fluorophore.

## 2. Materials and Methods

In this section, we describe our proposed simplified optical filtering system, the 3D-printed structure of our inverted microscope, and the use of a low-cost laser for sample excitation at 405 nm for FITC labeling.

### 2.1. Simplification of the Fluorescence Filtering System

To avoid high costs of complete filter cubes used by conventional fluorescence microscopes, we propose a simplified filtering system based on a longpass filter and a laser signal with narrow spectral width. First, we describe how conventional fluorescence filter cubes work, which includes a barrier filter, a dichroic mirror, and an excitation filter. Then, we describe our simplified system, which includes a longpass filter and replaces the excitation filter and the dichroic mirror with a laser module.

#### 2.1.1. Operation of a Conventional Fluorescence Filter Cube

**Figure 1:**
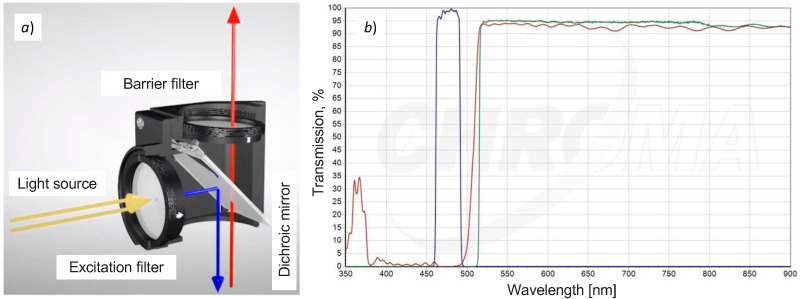
Filtering system of a conventional fluorescence microscope. (**a**) Operation of a fluorescence filter cube and (**b**) transmission spectra of the optical components of a longpass filter cube for FITC fluorophore.

Conventional fluorescence microscopy uses, for fluorochrome excitation, different polychromatic sources of light, e.g., mercury vapor lamps, halogen lamps, and white LED. All of these light sources produce white light, thus requiring a fluorescence filter cube, consisting of two types of filters (excitation and barrier filter), and a dichroic mirror (see Figure 1a). Fluorescence filter cubes can either have a longpass or a bandpass barrier filter [18]. The excitation filter is used to separate a narrow part of the white light source (see parallel yellow arrows in Figure 1a), which becomes the excitation signal for the fluorophore. In order to let the excitation signal reach the sample, a dichroic mirror (placed at 45°) reflects the excitation signal to the sample (see downwards blue arrow in Figure 1a). It then generates fluorescence in a different wavelength. Both the excitation and fluorescence signals are sent to the microscope’s ocular (see the upwards red arrow in Figure 1a), thus requiring a barrier filter to block the excitation signal, and let only the fluorescence signal pass.

Figure 1b shows the transmission spectra of the optical components of a longpass filter cube for the FITC fluorophore (excitation filter 480/30, dichroic mirror 505, barrier filter 515, and filter diameter 25 mm). We can observe a reduced spectral bandwidth excitation signal of around 30 nm generated by the excitation filter (see the blue curve between 450 nm and 500 nm in Figure 1b). The red curve shows the spectral behavior of the dichroic mirror, and the green curve shows the longpass filtering of the excitation signal, i.e., it makes the barrier filter completely transparent to the fluorescence signal.

#### 2.1.2. Using Low-Cost Laser Source to Increase the Optical Excitation Signal

To reduce the cost of sophisticated optics for the light filtering of a fluorescence filter cube, we need to reproduce a similar longpass excitation signal filtering as described before. The key idea of our approach is to replace the combination of (a) the white light excitation source, (b) the excitation filter, and (c) the dichroic mirror with a single low-cost laser source. The principle is that the laser has a very narrow spectral bandwidth (of around 5 nm), thus avoiding the need for the excitation filter (which was used to reduce the spectral bandwidth of the white light source). Furthermore, the dichroic mirror is not needed any more because the sample is directly excited by the laser beam. In addition, the intensity of the excitation laser signal is orders of magnitude higher than any white light source, thus increasing the fluorescence signal significantly. Finally, in our approach, the use of a longpass barrier filter remains the same as in the conventional fluorescence filter cube because we need to block the excitation signal while letting the fluorescence signal pass through.

**Figure 2:**
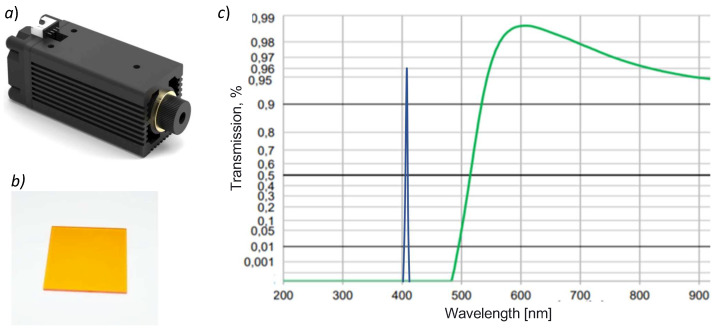
(**a**) The 405 nm laser module, (**b**) the longpass OG515 filter, and (**c**) the transmission spectra of the OG515 filter in green and the emission peak of the 405 nm laser module in blue.

Figure 2a shows the continuous focusable low-cost CW laser module that we used, which has the following characteristics: wavelength = 405 nm, voltage = 12VDC, current = 280 mA, optical power = 600 mW, PWM = 20 kHz–50 kHz/3.3VDC–12VDC, radiator size = 3 × 6 cm, and weight = 116 g.

Figure 2b shows the standard longpass OG515 filter used in our simplified filter system. The size of this filter is 25.4 × 25.4 × 3 mm and its transmission spectra are shown in Figure 2c, and were obtained using SCHOTT’s Interactive Filter Diagram [19]. The reached blocking ratio of the OG515 filter is five orders of magnitude between 200 and 480 nm (according to the filter’s datasheet [20]). We can observe that the longpass OG515 filter completely absorbs the laser excitation signal at 405 nm, regardless of the illumination angle. This confirms that our simplified laser-based filtering system is analogous to the one of the conventional fluorescence filter cube shown in Figure 1c, but the cost of the proposed system is up to an order of magnitude cheaper. Note that our device is not intended for single-molecule sensitivity (useful for antibody detection), which requires a blocking excitation of at least eight orders of magnitude.

### 2.2. The Design and Structure of the Inverted Microscope

Once the simplified low-cost laser-based longpass filter system was defined, we needed to find the most adapted structure, both to hold the filtering system and to allow for direct sample excitation with the laser beam (to avoid the need of a dichroic mirror). We found that an inverted microscope scheme fulfilled these requirements. Therefore, we used, as baseline, the structure of a non-fluorescence inverted smartphone-based microscope from [21], which was used for tuberculosis diagnosis. We adapted the structure to include the laser module on top of the sample holder to increase the magnification (from 100× to at least 400×), to integrate the longpass filter within the optical scheme, and to add X-Y-Z stage of samples.

**Figure 3:**
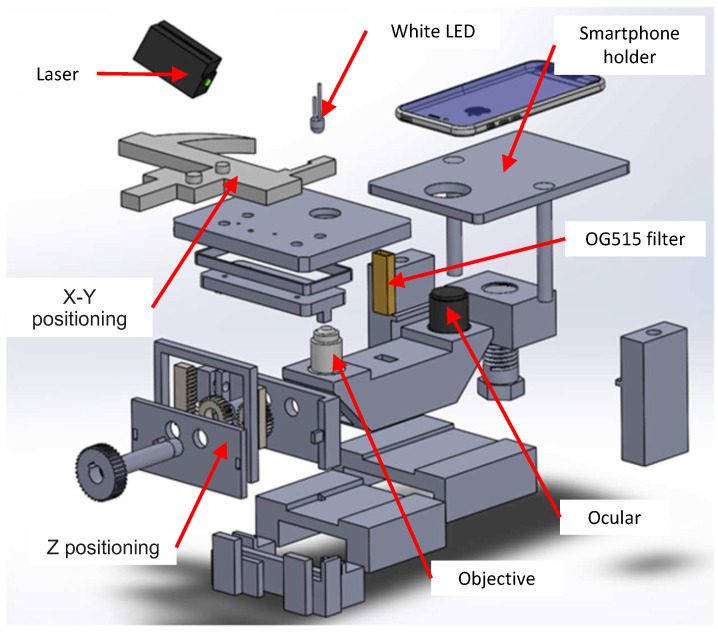
Block diagram of the 3D-printed laser fluorescence inverted microscope.

Our proposed inverted microscope consists of a continuous focusable 405 nm laser, a long pass OG515 filter, a high bright white LED, an optical ocular, an objective lens, a mechanical stage for biological microscope, and a 3D-printed structure, which includes a sample positioning system (X-Y and Z) and a smartphone holder (see Figure 3). As first-surface mirrors, we used a simple aluminium reflective surface recycled from old and damaged hard disks. All of the optical and mechanical components are inexpensive and can be easily found.

**Figure 4:**
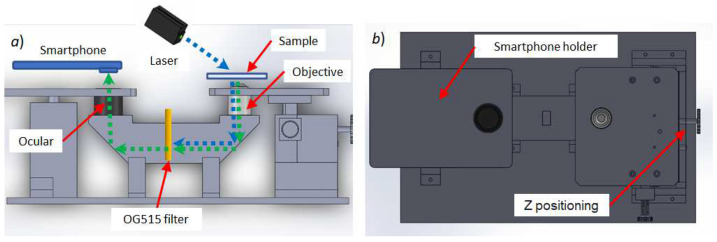
Three-dimensionally printed designed scheme. (**a**) Side view design showing the simplified longpass filtering system, and (**b**) top view design, where the smartphone is positioned, and the Z-positioning gear.

The optical structure and operating principle of the inverted microscope and our simplified filtering system is shown in Figure 4a. Once the sample is positioned, the laser beam directly excites the sample from the top. Both the excitation signal (blue dashed arrow) and the fluorescence signal (green dashed arrow) cross the objective lens, and are reflected by a first first-surface mirror positioned at 45°. Both signals reach the longpass OG515 filter, and the excitation signal is blocked. The filter lets the fluorescence signal reach the second first-surface mirror and cross the ocular, where the resulting image is amplified. The camera of the smartphone is positioned on the hole of the smartphone holder (see Figure 4b). The smartphone therefore captures the image with the fluorescence emitted by the sample labeled with a fluorophore, and the operator can directly visualize it and perform the diagnosis. The smartphone camera does not need a precise alignment with the pupil of the ocular since the type of disease diagnosis requires only observing an approximate shape of the microorganisms, but not any morphological details. Such alignment is good enough, even if it might generate a slight distortion of the image.

Note that the LED white light is not used for sample excitation, but only for initial sample illumination, e.g., for positioning the sample and helping with the focusing through the X-Y and Z positioning system. Thus, the operator must first turn ON the white LED light and focus the image before turning ON the laser module.

Our positioning system includes a conventional X-Y stage for microscopes that was adapted to the 3D-printed structure. For the Z positioning, a 3D-printed system of gears was designed to allow for coarse grain vertical movements of the piece where the objective lens is placed. Once the Z coarse adjustment was made through the 3D-printed gear, we used the objective lens’s screw for fine grain focusing. This allows the microscope operator to feel almost no difference compared to a conventional microscope, hence also reducing the costs of using a high-precision Z stage.

**Figure 5:**
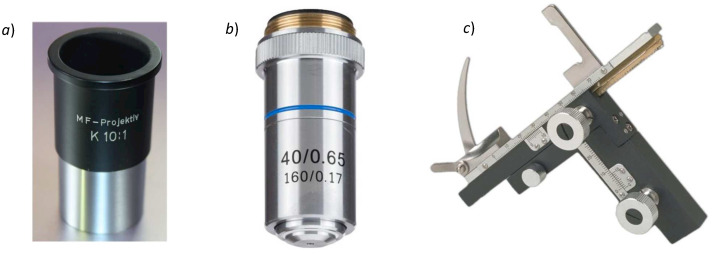
Optical and opto-mechanical components. (**a**) Optical ocular ZEISS MF-projektiv K 10×, (**b**) achromatic objective lens OMAX with 40× of magnification and 0.65 of numerical aperture (NA), and (**c**) OMAX X-Y microscope stage.

For an adequate magnification for disease diagnosis that is typically used in fluorescence microscopy, we used an ocular objective and an objective lens with 10× and 40× magnification, respectively (see Figure 5a,b). Both objectives were assembled in the 3D-printed mechanical structure. The numerical aperture (NA) of the objective lens in Figure 5b is 0.65, allowing us to resolve a structure of approximately 1 μm. The total magnification of our inverted microscope is 400×.

For the handling and positioning of the samples in the microscope, a conventional X-Y stage was integrated in the 3D-printed structure. Figure 5c shows the X-Y mechanical stage for the biological microscope.

### 2.3. Proposed Laser Excitation for FITC Labeling

FITC is a synthetic organic dye that eases the bio-conjugation with proteins (immunoglobulins) without interfering with their biological function. For several serological tests based on fluorescence labeling, FITC in one of the most commonly used fluorophores [14,15] due to its high absorption, excellent fluorescence quantum yield, affordable cost, and good water solubility [16].

FITC has an excitation absorption peak close to 495 nm [22,23,24]. For conventional fluorescence microscopy, the excitation is provided by a fluorescence filter cube 470/40 (where 480 nm is the peak of transmission with 30 nm of bandwidth). FITC is an important fluorophore, not only for fluorescence microscopy, but also for confocal microscopy and flow cytometry [25,26], where the main emission wavelength of the light sources (fluorescence filter cube 470/40 and argon ion laser) are also close to the 495 nm absorption peak (480 nm and 488 nm), respectively.

**Figure 6:**
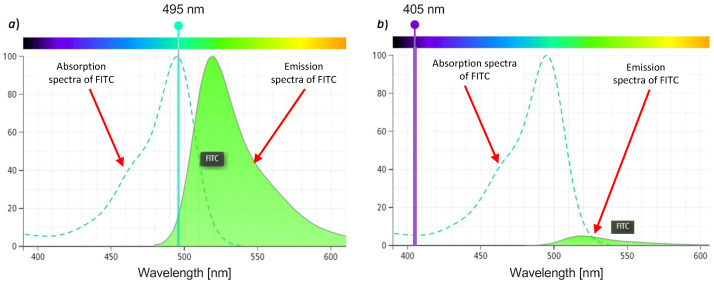
Absorbance and emission spectra of FITC fluorophore. (**a**) Excitation at 495 nm and (**b**) excitation at 405 nm.

Figure 6 shows the absorbance and emission spectra of the FITC fluorophore obtained with the DB Spectrum Viewer [27] simulator with two different excitation wavelengths. Figure 6a shows the maximum excitation wavelength for FITC at 495 nm, which results in the highest fluorophore absorption band (the dotted light blue curve). Therefore, the closer the wavelength of the excitation source is to the peak of the dotted curve, the higher the absorption will be, and, consequently, the higher the fluorescence emission. Note that the reduction in the emission spectra in the simulated Figure 6b is caused by the low absorption of the excitation signal and not by any bleaching effect.

For our low-cost inverted microscope, we propose the use of the laser excitation at 405 nm. Therefore, we can observe in Figure 6b that the absorption of FITC is rather low, and, consequently, the fluorescence emission will be lower.

However, since we are using a focalized laser source with a very narrow spectral bandwidth and a large number of emitted photons, this compensates for the low-level absorption of FITC. Our hypothesis, therefore, is that, through the use of the low-cost laser source, even though it is not at the peak of the FITC absorption band, we will be able to obtain a fluorescence value of sufficient intensity to clearly observe that signal.

In the next section, we show how this hypothesis was validated by testing microbeads labeled with three different fluorophores and, later, with real biological samples.

## 3. Results

In this section, we present the main results, which include the fully functional inverted microscope, the smartphone’s app used to process and visualize the images, the validation of the proposed filtering system with three different fluorophores, and the fluorescence images obtained with real Chagas parasites and ANCA granules in neutrophil cytoplasm, labeled with FITC with the 400× magnification. We also include the total cost of the proposed inverted microscope.

### 3.1. Assembling of a 3D-Printed Structure

**Figure 7:**
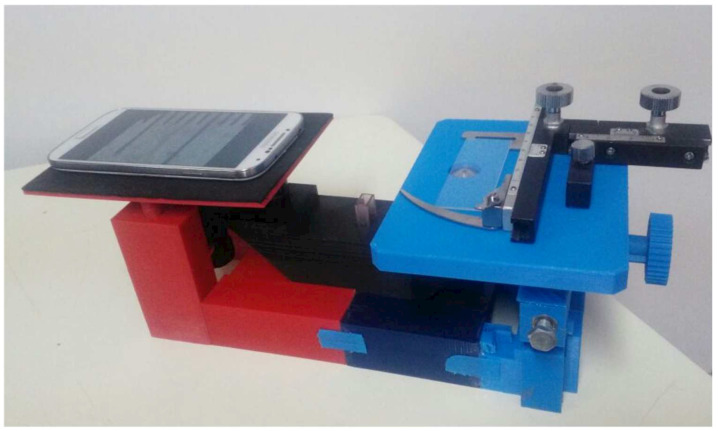
The 3D-printed structure of our smartphone-based inverted laser fluorescence microscope.

The proposed device was 3D-printed in a Flashforge Creator Pro 3D printer with a precision of 0.1 mm and the use of a PLA filament (as shown in Figure 7). The STL files of the 3D-printed structure are available as Supplementary Material. The piece containing all of the optical elements of the filtering system (objective lens, OG515 filter, ocular, and mirrors) was assembled in a compact box, printed with black PLA filament and with a higher density to minimize the interference of the external light.

The device consists of an inverted microscope structure and a smartphone. The smartphone is positioned on the structure’s ‘smartphone holder’ (with the turned-on camera). The sample slide is positioned in the ‘sample holder’, and the X-Y stage moves the slide to the correct position. The X-Y positioning system is a conventional X-Y microscope stage clip, allowing the operator to handle samples in a standard way. The images can be observed directly on the smartphone’s screen. The user only needs to turn ON the white LED and adjust the focus for the visualization of the magnified image. Then, to obtain a fluorescence image of the samples, the laser module needs to be turned ON and the LED needs to be turned OFF.

Our developed microscope is small (30 × 35 × 14 cm), robust, light-weight, and portable, requiring only electric power to operate the laser module.

### 3.2. Smartphone Processing and Optical Magnification of the Microscope

We developed an Android app (MicroscopeUPB) to capture, process, and store the images and videos generated by the microscope. The data are organized using an embedded database. The app allows the user to control the autofocus and lock it according to the user’s requirement. Since the smartphone’s camera captures the 400× magnified image directly from our microscope’s ocular lens (placed in the hole of the smartphone’s holder), the image displayed on the screen is shown as a circle that does not cover the full screen, and therefore is not very practical. The app, therefore, applies an automatic adjustment to the obtained image, so as to fit the ocular lens’s circle in the entire screen. This is carried out by cropping the circle and applying a 1.5 × magnification with digital zoom.

**Figure 8:**
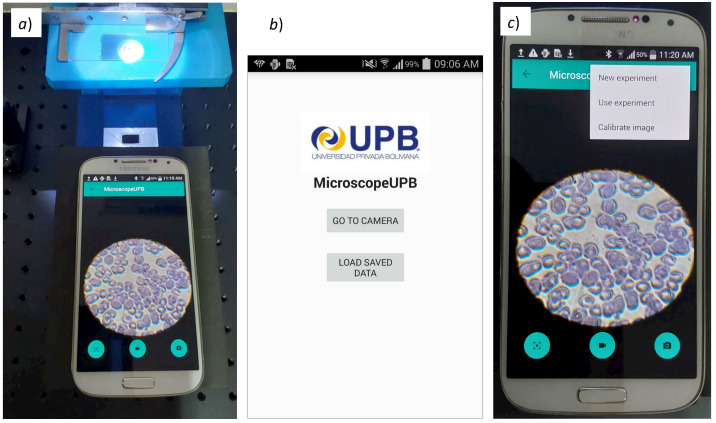
The Android app “MicroscopeUPB” for image capture and processing in operation. (**a**) Calibration of positioning; (**b**) main screen of the app; (**c**) capture options for experiments.

Figure 8a shows the inverted fluorescence microscope and the app in operation; Figure 8b shows the main screen with the options to capture the images and view previously stored experiment data; and Figure 8c shows the capture options for different experiments.

**Figure 9:**
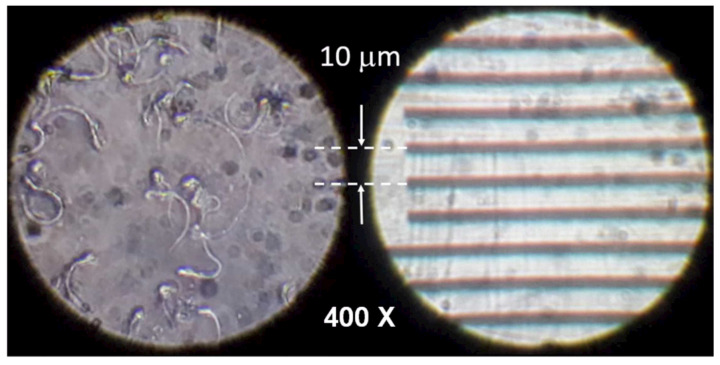
Effective magnification of the developed inverted microscope.

To validate the magnification of the device, we used a stage micrometer microscope calibration slider. Figure 9 shows the lines of the 10 μm calibration slider (on the right side) compared with the image captured with our microscope showing T. cruzi parasites (on the left side). This comparison confirms that our device has at least a 400× magnification, which is similar to the one obtained with conventional microscopes. The image was captured with a 13M pixel smartphone camera, which, combined with the 0.65 numerical aperture (NA) of the objective lens, allows for observing microorganisms of about 1 μm thickness, as shown in Figure 9. Note that the image was captured without using the digital zoom of the smartphone camera and only the white LED light was used to capture the image.

### 3.3. Validation of the Simplified Filtering System

**Figure 10:**
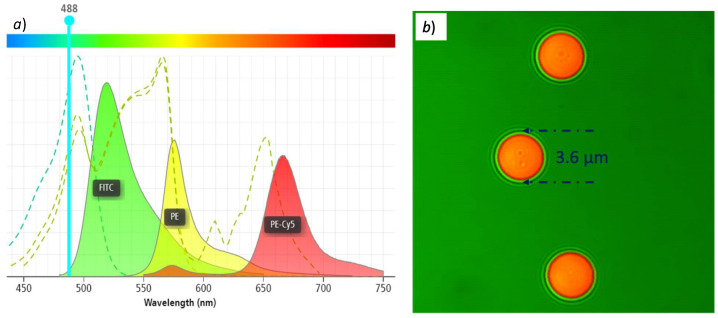
(**a**) Absorption and emission spectra of FITC, PE, and PE-Cy5 fluorophores under 488nm excitation; (**b**) ample calibration microbeads for flow-cytometry.

Our initial hypothesis was that we could use a laser excitation source at 405 nm instead of a standard wavelength of 488 nm, which is commonly used for a large variety of fluorophores in diagnostic techniques with fluorescence microscopy. Figure 10a shows the absorption and emission spectra for the FITC, PE and PE-Cy5 fluorophores, under an excitation at 488 nm. We can clearly see that, with that wavelength, all three fluorophores have high absorption curves and are close to their absorption peaks. Again, these peaks can be obtaining by using different types of light sources, but different and costly fluorescence filter cubes are needed.

To validate our hypothesis, we need to show that, using a laser with a different excitation signal (at 405 nm), thanks to the intense radiation and our simplified filter system, we are able to observe samples labeled with those fluorophores. For this, we used calibration microbeads that are normally applied for the verification and normalization of fluorescence intensities in flow cytometers. Figure 10b shows an example of such microbeads [28], which can be labeled with the desired fluorophore upon request. The typical dimensions of these microbeads are in the range of 2 μm to 6 μm, which require a magnification of 400× to be observed properly.

**Figure 11:**
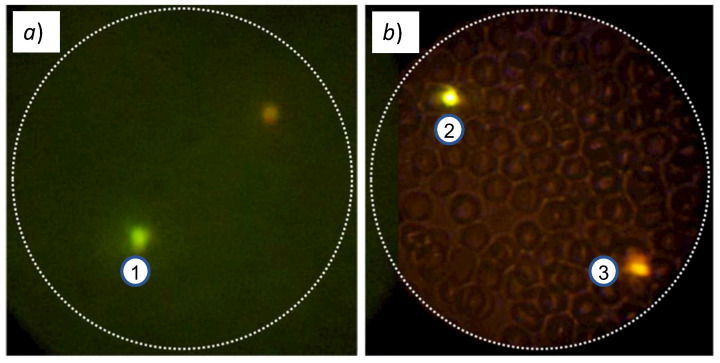

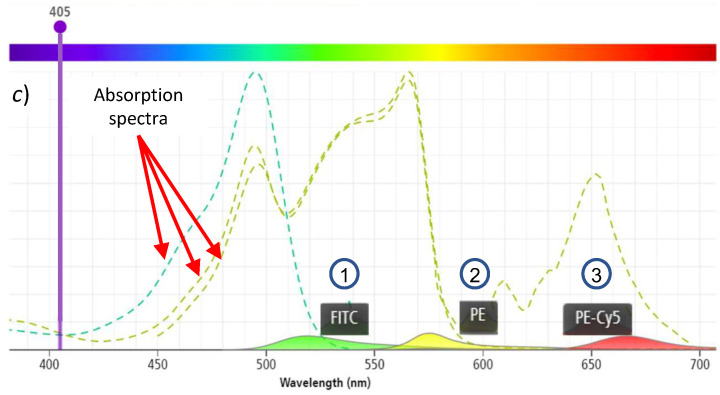
(**a**) Captured image of fluorescence of microbead labeled with FITC fluorophore; (**b**) fluorescence of microbeads labeled with PE and PE-Cy5 fluorophores; (**c**) absorption/emission spectra of microbeads under 405 nm laser excitation. The beads are in different focal planes, resulting in apparently deformed and fuzzy shapes.

We used our inverted fluorescence microscope to experimentally observe the fluorescence of microbeads labeled with FITC, PE, and PE-Cy5 fluorophores. The microbeads are present in a liquid solution that was put in a glass slide that was positioned in the X-Y stage, and the laser was turned ON. After the correct focusing and positioning, we were able to observe all three fluorophores. Figure 11a,b show the obtained fluorescence images of the 3.6 μm microbeads labeled with ➀ FITC, ➁ PE, and, ➂ PE-Cy5. As shown in Figure 11c, the emission peaks of these spheres, with a 405 nm excitation source, correspond to 519 nm (green color), 576 nm (yellow color), and 665.8 nm (red color), which are all in the visible part of the electromagnetic spectrum. Thanks to the intensity of the laser that we used, even though the emission curves are rather low, the obtained fluorescence images are visible with our device. This confirms that our simplified filter system works correctly, even with a non-optimal emission source.

### 3.4. Validation of Our Approach with Real Biological Samples Labeled with FITC

We validated our inverted fluorescence microscope by capturing images of real biological samples and comparing them with those obtained with a commercial conventional inverted microscope. For the validation, we chose samples of T. cruzi parasites and granules of neutrophil cytoplasm, both labeled with the FITC fluorophore.

#### 3.4.1. Validation with Chagas Infectious Disease

The epimastigotes forms of T. cruzi parasites were fixed on glass plates and were indirectly labeled using anti-human immunoglobulin IgG+IgM-FITC conjugate (Vircell S.L., Granada, Spain, Chagas IFA IgG-IgM Kit Ref. PCHAG). The labeling process was carried out following the kit supplier’s instructions. For T. cruzi staining, we used pooled human serum control from chronic Chagasic and non-Chagasic patients with 1:40–1:80 dilutions. Briefly, after sera dilution, 20–25 μL of each sample was placed in each of the slide wells and incubated for 30 ± 5 min at 37 °C. After two 5 min washes, they were incubated for 30 min with 25 μL/well with the respective FITC-labeled conjugate in Evan’s blue. After a new wash, the slides were mounted with buffered glycerin under coverslips for microscopic observation.

To compare the images obtained with our device, we used a commercial conventional fluorescence Motic BA410E microscope [29] with a magnification of 400×, using Eyepieces130N-WF 10×/22 and Objective EC-H Plan Achromats 40×/0.65. We used the same samples of T. cruzi parasites labeled with FITC fluorophore with both devices.

**Figure 12:**
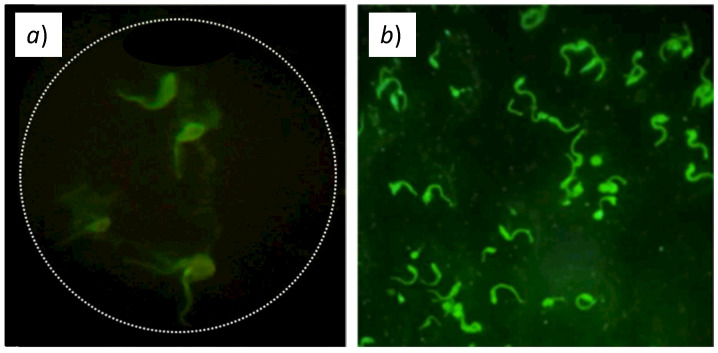
Fluorescence images of T. cruzi parasites labeled with FITC fluorophore, obtained with (**a**) our inverted fluorescence microscope; (**b**) a commercial Motic BA410E fluorescence microscope.

Figure 12a shows the image captured with our device, whereas Figure 12b shows the images captured by the commercial microscope. We can clearly see that our microscope is capable of identifying the T. cruzi parasites. We can observe that the commercial microscope has more intensity, which is due to the use of the optimal excitation signal. The images obtained with our device are slightly less intense because the use of the laser rapidly degrades the fluorophore due to the intense focalized laser beam. An immediate bleaching effect results in the total degradation of the fluorophore after only a few seconds of exposure. We can also see that the image obtained with our device shows bigger T. cruzi parasites (even though both microscopes have 400× magnification). This is due to a digital zoom made by our smartphone app to fit the ocular lens output on the screen.

#### 3.4.2. Validation with ANCA Non-Communicable Disease

**Figure 13:**
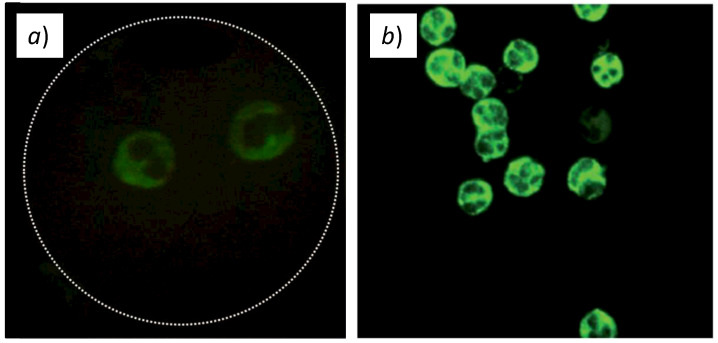
Fluorescence images of granules in neutrophil cytoplasm labeled with FITC fluorophore obtained with (**a**) our inverted fluorescence microscope; (**b**) a commercial Motic BA410E fluorescence microscope.

ANCA is a type of autoimmune disease that causes vasculitis. ANCA stands for anti-neutrophilic cytoplasmic autoantibody. Our device was also tested by visualizing neutrophils indirectly labeled with FITC-labeled anti-human IgG conjugate to find auto-antibodies against proteins of the cytoplasmic granules (Inova Diagnostics Inc., San Diego, CA, U.S., NOVA Lite^©^ ANCA Kit, Ref. 708290). For the labeling of neutrophils, undiluted ANCA-positive and negative controls from the kit were used. As for the T. cruzi sample preparation, we followed the kit supplier’s instruction and put the samples in the slides for observation. Figure 13a shows the image captured with our device, whereas Figure 13b shows the images captured by the commercial microscope.

Overall, we can confirm that our low-cost inverted fluorescence microscope is capable of capturing similar images from real biological samples labeled with FITC, and, therefore, can be used for disease diagnostic and biomedical applications.

### 3.5. Cost of the Proposed Low-Cost Inverted Fluorescence Microscope

The total cost of our proposed low-cost fluorescence microscope is around USD 290, which makes it affordable for low-income settings. Table 1 shows the list of components that are required to build the device, along with their cost. Note that the cost of the two components of our simplified longpass filtering system (i.e., the laser module and the OG515 longpass filter) does not exceed USD 130, which is largely less expensive than a commercial fluorescence filter cube, whose price is around USD 1000.

## 4. Discussion

Our initial hypothesis was that it was possible to build a low-cost fluorescence microscope by replacing the optical scheme of costly fluorescence filter cubes with a simplified filter system based on a laser and a cheap longpass barrier filter. Another hypothesis was that it was possible to reach a high enough magnification (400×) and use a smartphone to visualize the fluorescence of real biological samples for the diagnosis of diseases. Both of these hypotheses were totally validated and confirmed with the obtained results.

We also demonstrated that our approach works, even though the low-cost laser source with 405 nm wavelength is not the optimal one to reach the maximum of the 495 nm absorption peak of the FITC fluorophore. This was confirmed, during our validation with the calibration microbeads, by using two other fluorophores (PE and PE-Cy5) with different absorption peaks. This opens up the possibility of using other laser excitation sources with different wavelengths closer to the peak of the fluorophore to be used. For example, we could use diode lasers with 488 nm wavelength that appeared recently, together with an OG530 filter to completely block the excitation signal. It is worth mentioning that the use of argon ion lasers (also at 488 nm wavelength) is not a low-cost solution, since these lasers, although they can provide a wavelength close to the peak of the fluorophore, are expensive (in the order of USD 3000). Our future work includes testing changes in the excitation source and filter. Consequently, changing the excitation wavelength close to the fluorophore peak will allow us to reduce the intensity of the excitation laser signal and therefore substantially reduce the bleaching effect on the samples.

### 4.1. Advantages and Disadvantages of Our Approach

We can summarize the advantages of our low-cost laser fluorescence inverted microscope as follows:

- The device is easily reproducible and affordable, just requiring a 3D-printer, a smartphone, and low-cost optical and mechanical components.
- The magnification of the device is at least 400×, which is compatible with requirements for disease diagnosis (the visualization of parasites and cells).
- The device works with FITC, the most commonly used fluorophore for disease diagnosis, but also works with other fluorophores.
- The use of a smartphone allows digital zoom to have a better visualization of the fluorescence images.
- The inverted optical scheme simplifies the positioning of the smartphone camera through the smartphone holder.
- The device is small, portable, and does not require special working conditions.
- The X-Y and Z positioning system allows the microscope operator to handle the samples as in a conventional fluorescence microscope.

We can also mention some disadvantages of our approach, mainly related to the use of laser source:

- The use of a Class III laser module requires special care (e.g., using special protective eyewear) and operator training. We mitigate this by adding a protection filter to the device to absorb the laser excitation signal and by reducing the distance of the laser to the sample as much as possible.
- The exposure of the sample under laser radiation causes the fluorophores to degrade rapidly due to the high laser intensity (the fluorescence disappears after some seconds in our tests). This issue can be mitigated by enabling the intensity control system of the laser module (which works with a PWM pulse controller), which requires some electronic control system to be developed. In addition, another possibility is to replace the laser module with another one, with a wavelength closer to the absorption peak of the fluorophore (e.g., a laser module with 488 nm wavelength for FITC). This option also requires a change in the longpass barrier filter to reflect this change. This modification is totally valid but may increase the cost of the device.

### 4.2. Related Work on Low-Cost Fluorescence Microscopy

The development of low-cost microscopes has attracted a large body of researchers [3,4,5,6,7,8,21], thus giving an affordable alternative for developing countries due to the high costs of conventional microscopes. In [30], more than 20 types of low-cost microscopes were analyzed and reviewed, where some of them use smartphones as their optical sensor. Furthermore, smartphone-based microscopy [2] has become an interesting alternative, enabling more compact, affordable, and flexible solutions compared to conventional microscopy.

Due to potential healthcare applications, such as the detection and diagnosis of diseases, low-cost fluorescence microscopy was also developed in recent years. Our work is grounded in previous work in fluorescence microscopy [8] and the development of a non-fluorescence inverted microscope [21]. In the first one, we used Rhodamine 6G dye both to filter the excitation signal of a laser at 532 nm and also to visualize the fluorescent particles of Rhodamine 6G. The microscope used a Raspberry Pi camera as an optical sensor and had only a 100× magnification. In the latter, the inverted non-fluorescence microscope used a smartphone to collect images and videos from the microscopy observation drug susceptibility (MODS) technique for tuberculosis diagnosis. The inverted optical scheme was important to allow for Mycobacterium tuberculosis colonies grown in liquid media. We borrowed this inverted optical scheme to simplify our laser-based filtering system. This microscope had only a 100× magnification, which was enough to identify large colonies of Mycobacterium tuberculosis cords in MODS cultures.

Table 2 gives an overview of related work on low-cost fluorescence microscopy. We can observe that only one fluorescence microscope (Miller et al. [9]) was applied to the diagnosis of a disease. This is mainly due to the limited magnification of the other fluorescence microscopes, which do not reach the required 400× magnification, thus only allowing for the visualization of fluorescent biological and non-biological elements (e.g., cells, liver tissues, microbeads, and particles). Compared to our device, the microscope by Miller et al. also prevents the use of a fluorescence filter cube, by using an inverted scheme, but uses two filters (excitation filter and emission filter), uses spare parts of a conventional microscope, and does not use a smartphone.

The fluorescence microscope by Hasan et al. [7] is basically a re-implementation of a fluorescence filter cube based on a low-cost commercial microscope with an integrated camera, a dichroic mirror, and two filters (excitation and barrier). This fluorescence microscope does not use a smartphone, requires a computer to operate, has only a 200× magnification, and does not have a positioning system.

Although Schaefer et al. [10] proposed a fluorescence microscope with an automatized X-Y stage, similar to our previous work [8], the microscope focuses only on showing the feasibility of capturing fluorescent images as a proof-of-concept.

Dai et al. [5] propose a fluorescence microscope that uses a smartphone and a dual functional polymer for magnification and signal filtering, thus avoiding a costly fluorescence filter cube. The microscope uses different laser diodes as excitation source and has a resolution smaller than 200×, hence limiting its application only to the visualization of tissues.

Zhu et al. [6] propose a smartphone-based inverted fluorescence microscope with 400× magnification that uses an LED as excitation source; however, it uses a fluorescence filter cube, making the price rise to USD 3000.

Although the microscopes by Kim et al. [3], Dai et al. [5], and Liu et al. [4] are all smartphone-based, they only use a magnification lens attached to the smartphone camera (instead of an objective and ocular lens), thus limiting the magnification (up to 200×) and making them unfeasible for disease diagnosis. In addition, they do not have any positioning stage.

Overall, we can see that, compared to related work, our low-cost inverted fluorescence microscope fulfils the necessary features for the real diagnosis of diseases and sample handling close to those used in commercial fluorescence microscopes.

## 5. Conclusions

We successfully designed, built, and tested a smartphone-based low-cost laser inverted fluorescence microscope for the diagnosis of diseases. Our device includes a simplified optical filter system (using a low-cost laser source and a cheap barrier filter), thus avoiding the expensive filter system used in conventional fluorescence microscopes. We validated our device with real biological samples of one infectious disease (Chagas) and one non-communicable disease (ANCA), both labeled with an FITC fluorophore. This opens up the possibility of applying our approach to any disease that uses an FITC fluorophore for labeling. Our device has a 400× magnification and only costs USD 290 due to 3D printing and the use of low-cost material and recycled and spare parts. We believe that a low-cost fluorescence microscope can help for the early diagnosis of diseases in countries with low-income settings, where the availability of conventional commercial fluorescence microscopes is scarce or nonexistent.

## Figures and Tables

**Table 1:** Breakdown costs of our low-cost laser inverted fluorescence microscope.

Component Used	Model/Specification	Estimated Cost (USD)
X-Y Stage ${}^{a}$	OMAX X-Y Mechanical Stage for Biological Microscope	20
Ocular lens ${}^{a}$	ZEISS MF-projektiv K 10×	20
Objective lens ${}^{a}$	OMAX Achromatic Biological Microscope Objective Lens 40×	30
Light source	White LED	1
Laser source	Focusable, 12 V, 600 mW, 405 nm CW laser module	100
OG515 filter	25.4 × 25.4 × 3 mm	29
Electrical components	N/A	20
3D-printed	PLA filament (1 kg)	20
Holder	Laser and LED holder	10
Opaque cap ${}^{a}$	Protective safety interrupter	40
Mirrors ${}^{b}$	Aluminium first-surface mirrors	0
	Total cost of proposed microscope	290

^*a*^ Spare part of conventional microscope, ^*b*^ Recycled from old hard disk.

**Table 2:** Related work on low-cost fluorescence microscopes.

Authors, Reference, Year	Smartphone Based	Mag.	Emission Source	Fluorophore	Type of Application	Cost [USD]
Miller et al. [9] 2010	No	400×	Blue LED and excitation filter	Auramine	Diagnosis of M. tuberculosis	240–480
Schaefer et al. [10] 2012	No	200×	LED ( 460 nm)	N/A	Visualization of fluorescent microbeads	150
Kim et at. [3] 2015	Yes	200×	LED ( 405 nm) and Blue laser ( 445 nm)	Carboxyfluorescein succinimidyl	Visualization of cells	N/A
Hasan et al. [7] 2016	No	200×	Flashlight and excitation filter (380–500 nm)	Alexa Fluor 488	Visualization of cancer cells	358
Ormachea et al. [8] 2017	No	100×	Green laser ( 532 nm)	Rhodaimine 6G	Visualization of fluorescent particles	620
Dai et al. [5] 2019	Yes	<200×	Laser diodes (365, 480, and 520 nm)	DAPI and Alexa Fluor 488	Visualization of human liver tissues	N/A
Zhu et al. [6] 2020	Yes	400×	LED ( 470 nm)	PARPi-FL	Visualization of swine esophagus tissue cells	3000
Liu et al. [4] 2021	Yes	20×	UV LED ( 285 nm)	Rhodamine B DAPI and CytoStain	Visualization of mice live cells	20–50
Our device	Yes	400×	Blue Laser ( 405 nm)	FITC	Diagnosis of Chagas and ANCA	290

## Data Availability

Not applicable.

## Supplementary Materials

The following supporting information can be downloaded at: https://www.mdpi.com/article/10.3390/bios12110960/s1. This includes all of the STL files used to 3D print the inverted microscope structure.

## Abbreviations

The following abbreviations are used in this manuscript:

ANCA	Antineutrophil Cytoplasmic Antibodies
CW	Continuous Wave
FITC	Fluorescein Isothiocyanate
IFA	indirect immunofluorescent-antibody
LED	Light Emitting Diode
PLA	Polylactic Acid
PE	Phycoerythrin
PE-Cy5	Phycoerythrin - Cyanine 5
UV	Ultra Violet
